# Biomimetics and Education in Europe: Challenges, Opportunities, and Variety

**DOI:** 10.3390/biomimetics6030049

**Published:** 2021-08-04

**Authors:** Olga Speck, Thomas Speck

**Affiliations:** 1 Plant Biomechanics Group @ Botanic Garden Freiburg, University of Freiburg, D-79104 Freiburg, Germany; thomas.speck@biologie.uni-freiburg.de; 2 Cluster of Excellence *liv*MatS @ FIT—Freiburg Center for Interactive Materials and Bioinspired Technologies, D-79110 Freiburg, Germany

**Keywords:** architecture, biomimetics, bioinspiration, education, equity, interdisciplinarity, training, STEM = science, technology, engineering, mathematics

## Abstract

Biomimetics is an interdisciplinary field of science that deals with the analysis and systematic transfer of biological insights into technical applications. Moreover, the development of biomimetic products helps to improve our understanding of biological concept generators (reverse biomimetics). What does this mean for the education of kindergarten children, pupils, students, teachers, and others interested in biomimetics? The challenge of biomimetics is to have a solid knowledge base in the scientific disciplines involved and the competency to be open-minded enough to develop innovative solutions. This apparently contradictory combination ensures the transfer of knowledge from biology to engineering and vice versa on the basis of a common language that is perfectly understandable to everyone, e.g., the language of models, algorithms, and complete mathematical formulations. The opportunity within biomimetics is its ability to arouse student interest in technology via the fascination inherent in biological solutions and to awaken enthusiasm for living nature via the understanding of technology. Collaboration in working groups promotes professional, social, and personal skills. The variety of biomimetics is mirrored by the large number of educational modules developed with respect to existing biomimetic products and methods.

## 1. Introduction

### 1.1. Status Quo

The Corona pandemic has dramatically shown us the importance of the preparation of teaching content in such a way that pupils and students can work on it themselves. As practical courses with technical exercises are increasingly required to gain knowledge and associated skills, the situation within education becomes precarious. In this context, and even in regular university and school settings, experiments on biomimetics are particularly well suited to impart scientific and technical knowledge and thinking. A variety of experiments on biomimetics can be performed with everyday resources (e.g., bowl of water, dishwashing liquid, plant leaves) and with simple safety precautions (e.g., safety goggles, apron, gloves). Another advantage of biomimetics is that findings from current research can be made rapidly available via simple practical experiments or thought experiments. Target groups for biomimetic modules include not only pupils and students, but also kindergarten children, visitors to extracurricular places of learning (e.g., science centers, zoological and botanical gardens), and lay people of all ages interested in biomimetics. Biomimetic modules can be used in primary, secondary, and tertiary education, and also for lifelong learning. We present, in Table 1, further background information concerning the state of education and educational development in the OECD (=Organisation for Economic Co-operation and Development), in the EU (=European Communion), and in Germany, particularly with regard to the natural sciences and to engineering.

The OECD’s Programme for International Student Assessment (PISA) is a worldwide study that measures the abilities of 15-year-olds to use their reading, mathematics, and science knowledge and skills to meet real-life challenges. PISA tries to be different from traditional assessments. Instead of measuring the knowledge that students can recognize, PISA assesses whether they “are able to extrapolate from what they know, think across the boundaries of subject-matter disciplines, apply their knowledge creatively in novel situations and demonstrate effective learning strategies.” [2], p. 3.

Moreover, studies in recent years have shown that a strong social selectivity exists in the education systems of many countries [3,4]. Non-academic children are disadvantaged from elementary school to doctoral studies because of social selection. In Germany, the so-called “Bildungstrichter” (translated into English: “educational funnel”) means that 21 out of 100 children of non-academic parents start university, 15 out of 100 attain a bachelor’s degree, eight out of 100 earn a master’s degree, and one obtains a doctoral degree. In contrast, 74 out of 100 children of academics begin university, 63 out of 100 earn a bachelor’s degree, 45 out of 100 attain a master’s degree, and 10 out of 100 earn a doctoral degree [3]. Insufficient learning support from the parental home leads to a threat to educational equity and equal opportunities for school-age children, a threat that is exacerbated in the Corona crisis by poor digital accessibility and a lack of contact with peers [5].

Apart from national education systems, education is a worldwide task that must be mastered internationally. UNICEF (=United Nations Children’s Emergency Fund) claims that all children have the right to go to school and learn, regardless of who they are, where they live, or how much money their family has. However, in 2018 worldwide 8.2% of primary-school-aged children, 15.6% of lower-secondary-aged children, and 35.2% of upper-secondary-aged youth were not in school at all [6]. The Global Education Coalition launched by UNESCO (=United Nations Educational, Scientific and Cultural Organization) brings together 175 members from the UN family, civil society, academia, and the private sector to ensure learning continuity. Since 1.5 billion students on Earth are affected by school and university closures due to the COVID-19 pandemic, UNESCO supports countries in their efforts to address learning losses and to adapt education systems [7]. Quality Education is one of the Sustainability Developmental Goals (SDG 4) established by the United Nations as part of the Agenda 2030. SDG 4 includes targets of free or equal access to quality education, lifelong learning opportunities, and education for sustainable development to be achieved by 2030 [8]. 

What conclusions can be drawn from the results of these various studies and programs? In order to ensure equity and equal opportunities in education, new educational content and educational standards have been established. Equity initiatives support STEM (=science, technology, engineering, and mathematics) programs throughout the educational chain. Since all areas of society are characterized by technical and scientific innovations and challenges requiring solutions, we need to participate professionally and socially in such interdisciplinary challenges. In recent years, the field of biomimetics, as a combination of biology and technology, has clearly been shown to make a significant contribution to education and training. Biomimetics is an interdisciplinary field of science that deals with the analysis and systematic transfer of biological insights into technical applications. Furthermore, in the process of reverse biomimetics, the development of biomimetic products might help to improve our understanding of biological models. Simple experiments facilitate access to independent working and train teamwork skills.

### 1.2. Motivation

In the present publication, we wish to show the potential of biomimetics for various target groups such as kindergarten children, pupils, students, teachers, and others interested in biomimetics and bioinspiration. Based on successful biomimetic developments, we highlight the challenges of finding a compromise between knowledge-based and competence-based educational standards. We also suggest that student interest in technology can be aroused via a fascination inherent in biological solutions developed during evolution and that enthusiasm for living nature can be awakened through the understanding of technology. In addition, we present a variety of educational modules developed with respect to biomimetic products.

## 2. Challenges: Combining Knowledge and Competence

In general, a distinction is made between knowledge-oriented and competence-oriented teaching and learning. Knowledge orientation is based on conventional curricula, is teacher-centered, and is a learning style with the goal of accumulating details (e.g., formula, vocabulary) that can be reproduced. Competence orientation, however, is based on educational standards, assessment, and evaluation, is student-centered, and is a learning style with the goal of acquiring skills (e.g., teamwork, communication, decision-making), appropriate attitudes, and an understanding of concepts in order to apply knowledge. In recent years, as a result of the unsatisfactory results of the PISA studies, a change in perspective has occurred from input-oriented (=knowledge-oriented) to output-oriented (=competence-oriented) thinking or, in other words, from “less details—but more competencies” [9]. 

**Figure 1:**
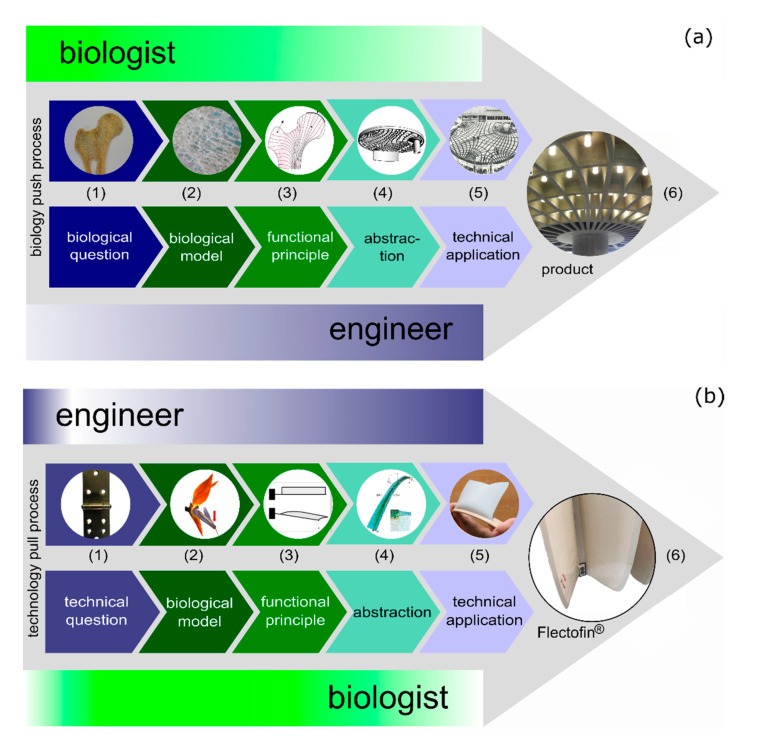
Biomimetic approaches. (**a**) Biology push process (=biomimetic bottom-up approach) of the bone-inspired concrete ceiling of the Old Zoology Lecture Hall at the University of Freiburg, Germany. (1) What makes bone a lightweight construction?; (2) trabeculae of the bone; (3) bone trabeculae align with main forces; (4) model of the ceiling; (5) ceiling in construction (© Bruno Krupp, Freiburg and University Building Authority (Universitätsbauamt) Freiburg).; (6) ceiling detail. (**b**) Technology pull process (=biomimetic top-down approach) of the façade shading system Fectofin^®^. (1) How to create a hinge-less motile shading system?; (2) perch of the bird-of-paradise flower; (3) torsional buckling; (4) finite element model (photo courtesy: Simon Schleicher); (5) simple physical model; (6) façade shading system Flectofin^®^.

In biomimetics, we need both knowledge and competencies. The development of a biomimetic product is a stepwise process. In an interdisciplinary approach, experts from various scientific fields (e.g., biology, chemistry, physics, mathematics, IT, design, architecture, engineering, materials sciences) analyze and systematically transfer biological insights into technical applications. In general, we distinguish between the biology push process (=biomimetic bottom-up approach), which starts with a biological research question, and the technology pull process (=biomimetic top-down approach), which starts with a question that formulates a technical challenge. After having selected a suitable biological model, the functional principle must be deciphered and translated into an engineering-compatible language. This abstraction step guarantees that not all details of the biological model are simply copied, but that only those elements of the biological model considered essential for the desired function(s) are assembled. Once the functional principle has been abstracted in the form of functional models, we can make construction plans, circuit diagrams, numerical models, analytical models, a demonstrator, and then a prototype. Ultimately, biomimetic products or methods can be produced for the market [10,11,12]. Figure 1 shows both biomimetic approaches by means of well-known examples.

Further biomimetic products developed in a biology push process are the self-cleaning surfaces that bear the trademark Lotus-Effect^®^ inspired by lotus leaves [13], the hook-and-loop fastener with the trademark Velcro^®^ inspired by the attachment system of hooks of burdock seeds and dog fur [14], and the drag-reducing riblet films on aircraft and riblet textiles for swimming inspired by sharkskin [15]. Well-known biomimetic products developed in a technology pull process are the façade shading system Flectofin^®^ inspired by the movement of the perch of the bird-of-paradise flower [16], the self-sealing coating for pneumatic systems of the Tensairity^®^ technology inspired by the wound sealing of the liana *Aristolochia macrophylla* [17], and aircraft winglets inspired by bird wings [18].

Knowledge from the natural sciences is a prerequisite for understanding the biological question, for selecting a suitable biological model, for performing appropriate experiments, and for discussing the results in the context of biomimetics. Knowledge from engineering sciences is necessary for understanding the technical challenge and for transferring the results obtained into a technical product. Competencies such as knowledge transfer and interdisciplinary thinking are needed to decipher the functional principle and to abstract it in the form of a common language understood by natural scientists and engineers.

The two biomimetic approaches are suitable for promoting students’ understanding of innovation processes as systematic step-by-step procedures. Thus, they learn to think and act in a way that combines natural sciences and technology. Moreover, the development of biomimetic products improves their understanding of biological concept generators. Within this so-called process of reverse biomimetics, the functional principles and abstracted models (typically including finite element modeling) are repeatedly evaluated in iterative feed-back loops and compared with the biological models leading to a considerable gain in knowledge. Through the collaboration of scientists from different disciplines, biomimetics is a pathway to interdisciplinary knowledge and competencies.

## 3. Opportunities: Facilitated Access to Biology and Technology

Biomimetics is highly suitable for teaching scientific and technical content in schools and universities for the following reasons. Since biomimetics is a new field, we have no need to fall back on traditions. On the contrary, new teaching content and modern topics can be offered in basic science subjects (e.g., biology and physics) or in newly created combinations of subjects. In 2007, for example, the State Baden-Württemberg, Germany, introduced into secondary schools the subject “Naturwissenschaft und Technik (=NwT = natural sciences and technology) in which biomimetics can make a significant contribution.

As has become apparent in recent years, brand-new results from biomimetics are highly likely to be quickly transferred to educational modules for all target groups. Because of the high demand from teachers, we published an educational module for the biomimetic facade shading system Flectofin^®^ while the R&D project was still running [16,19].

The study of biomimetic developments might arouse the interest of students in technology through the fascination inherent in biological solutions developed during evolution. Moreover, the enthusiasm of students for living nature might be awakened through an understanding of technology. Such an unorthodox entry into STEM subjects via biomimetics might reduce the reservations and fears that students have about quantitative biology (e.g., biomechanics, morphometrics) and technology (e.g., equations, figures). They can thus expand their understanding of technology (e.g., construction, function, production processes) and the basic sciences (e.g., biology, chemistry, physics, mathematics) and come to appreciate their importance in everyday life. Objects in our everyday life increasingly are based on models from living nature. We often do not realize this because no design language exists for biomimetic products. The presentation and developmental history of well-known examples such as the self-cleaning façade paint Lotusan^®^, the Velcro^®^ fastener, or barbed wire can encourage students to learn more about them. Table 2 presents the steps from a biological or technical question to a biomimetic product with respect to several educational modules that we have developed for various target groups, as shown in Table 3.

## 4. Variety: Education of Various Target Groups

Biomimetics is suitable for all age groups. Natural phenomena, technical relationships, and the development of biomimetic products can be interesting for kindergarten children, pupils, students, teachers, and pensioners. Educational modules for specific bio-mimetic products, such as the adhesive force of Velcro^®^ fasteners, the self-cleaning of surfaces, or the actuation of the Fluidic Muscle, are therefore available for the different target groups. A kindergarten child can observe the self-cleaning of various plant leaves. On the other hand, with regard to lifelong learning, the development of the façade paint Lotusan^®^ can be illustrated during a guided tour in a botanical garden by means of simple experiments on plant leaves and façade paint samples with or without the self-cleaning effect.

Within the framework of educational modules on biomimetics, students acquire personal skills (e.g., personal responsibility, perseverance and frustration tolerance while undertaking projects, personal initiative), professional competencies (e.g., interdisciplinary working and thinking, understanding of industrial production processes, understanding of innovation processes, and critical open-mindedness with respect to new technologies), and social competencies (e.g., ability to work in a team, communication skills, cooperation, and responsibility). 

Pupils and students learn to plan, carry out, evaluate, and record experiments independently. They exercise responsibility for the tasks assigned to them and experience team working. They also learn to apply functional principles by means of technical design. Since the close connection between biomimetics and sustainability is repeatedly discussed within such projects, dealing with these topics increases critical open-mindedness for sustainable technological development. In this context, students can broaden their view of biodiversity: every species that becomes extinct reduces the available models for a present or future challenge to humankind.

In recent years, a variety of meaningful new teaching models has occurred in various fields. With reference to the educational module “The bone-inspired ceiling” presented in detail in the Supplementary Material S1, we wish to concentrate here on the field of architecture. In a survey, 93% of students stated that they were informed about nature-inspired designs, and that their main motivation was “designing architectures in harmony with nature” [33]. Studio One at the University of Berkley (USA) established experiment-based and project-driven teaching bringing together students from various disciplines. Inspired by biological concept generators, they produced 2D sketches, small 3D models, 1:1 prototypes, and pavilions [34]. Other examples include the student projects on bioinspired pavilions, which have run for more than a decade in the Institute of Building Structures and Structural Design (ITKE) and the Institute for Computational Design and Construction (ICD) at the University of Stuttgart [35,36].

Table 3 gives an overview of the educational modules that we have developed for various target groups based on well-known biomimetic products and optimization methods. We developed most of the modules for German-speaking countries (Germany, Austria, and Switzerland), but currently we are also gradually making them available to English speakers. In all modules, we wish to raise awareness that biomimetic products are not simple copies of the biological model or analogies between biological and technical structures (e.g., suction cup), because analogies are merely the expression of the validity of the same basic physical, chemical, and mathematical laws in the living and technological realms and do not (per se) include bioinspiration. 

Rather, the focus of all of our modules is the transfer of the functional principle and an abstraction from the biological model to the technical application. The image of a turnstile best describes our didactic concept. The many details of the biological model are ordered, separated, and evaluated, and only those that form the basis for the creation of functional models, construction plans, circuit diagrams, analytical models, or numerical models are allowed to pass through. 

## 5. Quo Vadis?

In discussions about improvements in the education system, calls are immediately made for more money, more teachers, smaller class sizes, additional daycare centers, and more all-day facilities. Although such requests should certainly be considered, we should also take into account Hattie’s meta-study [37] in which the results of over 100,000 individual studies involving 300 million students around the world have been compiled. The study ranks 252 influencing factors according to their effects on student learning and achievement at school. The effects are measured by effect size, which is on average 0.4, a metric that represents one year of growth per school year for a student. Anything above 0.4 would have a greater positive effect on student learning. The three best places are occupied by “teacher estimates of achievement” (effect size = 1.62), “collective teacher efficacy” (1.57), and “self-reported grades” (1.33). Negative influences arise from “summer vacation” (−0.02), “television” (−0.18), and “moving between schools” (−0.34). Reduction of class size has only a small positive impact (0.15). Mathematics programs (0.58) and science programs (0.56) have the potential to accelerate the impact on student achievement. Conceptual change programs (0.99), in which teachers help their students build an understanding of complex scientific concepts by breaking them down into their component parts, are even more influential, depending on their students’ level of knowledge and development [37,38]. Within mathematics and science programs, biomimetics can make a significant contribution to student learning through its interdisciplinary nature. In addition, biomimetic topics can be broken down into single concept elements according to the level of intellectual development of the students. With regard to the understanding concepts, one particularly interesting and topical aspect is the relationship between biomimetic developments and their contribution to sustainability.

In principle, the subject of biomimetics has great potential in the development of sustainable materials, structures, and technologies, but sustainability is not an automatic by-product generated by the idea flow from biology to technology. With respect to both conventional and biomimetic developments, contributions to a more sustainable future can only be ensured if a particular ethos and respect for nature accompany the technological ambitions of the practice [39]. In the sense of conceptual change programs, biomimetics can be divided into its individual development steps according to the relevant biomimetic approaches (Figure 1), and the concept of sustainability can be considered under environmental, economic and social aspects. In recent years, we have carried out various sustainability assessments of selected biomimetic products and biomimetic methods in order to qualitatively and quantitatively analyze their contribution to sustainability [40,41,42,43]. These assessments include the bone-inspired ceiling of the Old Zoology Lecture Hall, a lightweight construction at the University of Freiburg, Germany (Figure 1a, Supplementary Material S1). We have compared its contribution to sustainability qualitatively with that of a solid slab [41] by using a straightforward sustainability assessment. Additionally, we have carried out a quantitative assessment with two conventional lightweight ceiling structures, namely a hollow article ceiling and a pre-stressed flat ceiling. Life cycle assessment and potential social aspects show comparable results. The biomimetic ribbed ceiling, however, is 2.2 times more costly because of the complex formwork [43]. Comparative sustainability studies of conventional and biomimetic developments are intended to encourage students to adopt a critical attitude with respect to innovations in their everyday lives.

In our view, technical solutions based on biological models enable pupils and students to gain knowledge and develop competencies that make them competitive, that allow them to participate actively in society, and that ensure their solid vocational training and continuing education. However, school and university education should not be considered merely as a “stock of knowledge” that lasts a lifetime but should prepare students for lifelong learning and further education. Extracurricular places of learning (e.g., science centers, zoological and botanical gardens) play a major role in lifelong education, as they offer exhibitions, guided tours, lecture series, and many opportunities for broadening the mind for all age groups. A questionnaire survey in the National Museum of Nature and Science in Tokyo (Japan), where a biomimetics exhibition was held, revealed that expectations about biomimetic applications are different between generations. Younger-aged visitors expected medical applications and middle-aged visitors expected environment and energy application [44]. Topics on biomimetics encourage visitors to understand systems from the perspective of the complex interrelationships of animate and inanimate nature and technology as based on the methods of the natural sciences and technology and, ultimately, to form a holistic view. Thus, biomimetics can contribute to higher standards, better teaching, and individual support, and to the change from equal opportunities in education to educational equity.

## 6. Conclusions

In summary, ten arguments support the valuable contribution that biomimetics can make to education:

1. Enthusiasm for technology is aroused through access via biological models.
2. Enthusiasm for living nature is aroused through technical challenges and solutions.
3. Up-to-date scientific research results can be presented in a timely manner in educational modules that have been newly developed and that are easy to understand and to perform.
4. Students acquire personal skills: personal responsibility, perseverance and frustration tolerance in projects, and personal initiative.
5. Students acquire professional competencies: interdisciplinary working and thinking, understanding of industrial production processes, understanding of innovation processes, and critical open-mindedness for new technologies.
6. Students acquire social competencies: ability to work in a team, communication skills, cooperation, and responsibility.
7. Awareness of biodiversity is increased.
8. The discussion of “biomimetics and sustainable development” is encouraged.
9. Young scientists are recruited: the variety of topics and activities in the field of biomimetics is a pathway to interdisciplinary knowledge and competence.
10. Lifelong professional qualification is enhanced through the interdisciplinary approach of biomimetics.

## Figures and Tables

**Table 1:** Education in figures. Comparison of average values in the educational sector in the OECD (=Organisation for Economic Co-operation and Development), EU-28 (=28 member states of the European Communion until January 2020), and Germany [1,2].

Topic	OECD	EU-28	Germany	Reference
Expenditure on educational institutions in 2017 as % of GDP (=Gross Domestic Product)	4.9%	—	4.9%	[1] Table C2.1
Educational participation in 2018	14 years	—	15 years	[1] Table B1.1
Average class size for primary education in 2018	21.1	—	21.0	[1] Table D2.3
Average class size for secondary education in 2018	23.3	—	23.9	[1] Table D2.3
Graduates of tertiary education in 2018 in natural sciences and mathematics	5.5%	—	9.1%	[1] Table B5.2a
Graduates of tertiary education in 2018 in engineering	14.3%	—	21.4%	[1] Table B5.2a
25- to 64-year-olds participating in lifelong learning in 2019	—	11.3%	8.2%	[1] Table A8-EU
PISA 2018: skills in reading	487		498	[2]
PISA 2018: skills in mathematics	489		500	[2]
PISA 2018: skills in natural sciences	489		503	[2]

**Table 2:** Description of the step-by-step development of selected biomimetic examples.

Biomimetic Approach	Question	Biological Model	Functional Principle	Abstraction	Biomimetic Product/Method
	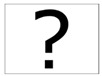	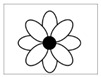	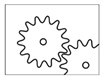	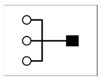	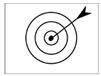
Biology push(Bottom-upapproach)	What makes self-cleaning leaves?	Plant leaves	Water repellency: water contact angle > 170° and contact area of droplet ≈ 0.6%	1. Micro- and nano- rough surface 2. Hydrophobic surface 3. Surface tension of water	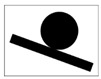 **Lotus-Effect^®^**
Biology push (Bottom-up approach)	How do burrs stick to animal fur?	Burdock seeds together with animal fur	Reversible and random attachment:elastic hooks cling to fur or fabrics	1. Hook tape with thick hooks 2. Loop tape with many small loops	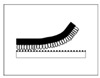 **Velcro^®^**
Technology pull (Top-down approach)	How to lift a mass?	Skeletal muscle	Cylinder surrounded by spirally netted fibers with variable fiber angle	Fiber angle < 54.7°: pressure-tight hose shortens when filled with compressed air	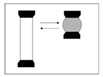 **Fluidic muscle**
Biology push (Bottom-up approach)	How does a fish fin generate propulsion force?	Fish fin	Self-adaptive shape	Isosceles acute-angled triangle of two bending flexible longitudinal struts and flexibly connected cross struts	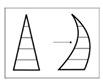 **FinRay Effect**
Biology push (Bottom-up approach)	What makes bone a lightweight construction?	Internal bone structure	Bone trabeculae along the main force lines	1:20 model, stress tests analyzed with photoelasticity	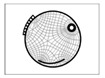 **Lightweight ceiling**
Technology pull (Top-down approach)	How to quickly seal a leak in a pneumatic system?	Wound sealing in liana stems	Self-sealing cells squeeze into the rupture	Internal polyurethane foam coating with closed pores rapidly seals fissures	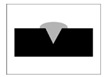 **Self-sealing cells**
Biology push (Bottom-up approach)	How do external cracks seal in succulent leaves?	Wound sealing in succulent leaves	Hydraulically and mechanically driven leaf deformation until the wound edges meet	Polymer with shape-memory effect	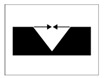 **Self-sealing by deformation**
Technology pull (Top-down approach)	How to create a hinge-less flapping system?	Movement of the perch of *Strelitzia* flowers	Torsional buckling	Finite element modeling	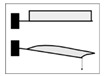 **Flectofin^®^**
Technology pull (Top-down approach)	How to optimize notch shapes?	Growth processes of trees	Trees respond to notches through adaptive growth	Reinforcement of highly stressed outer areas of components until a shape without stress peaks is obtained	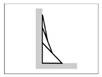 **Tensile triangles, Computer Aided Optimization**
Technology pull (Top-down approach)	How to create lightweight structures?	Growth processes in bones	Adaptation to new loads by building up and removing bone material	Creating a lightweight design through removal of non-load-bearing areas	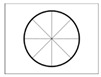 **Soft Kill Option**
Technology pull (Top-down approach)	How to find the optimal solution without knowing the target?	Evolutionary concepts	Reproduction, mutation, recombination and selection	Population-based optimization algorithms	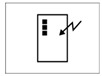 **Evolutionary algorithms**

**Table 3:** Educational modules of well-known biomimetic products and methods developed for various target groups. All modules have instructions for and evaluations of the experiments, model solutions, and explanations (abbreviated as “Hands-on experiments”).

Pictogram	Biomimetic Product or Method	Educational Module	Brief Description	Content	Target Group	Language [Reference]
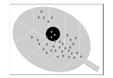	**Lotus-Effect^®^**	Self-cleaning leaf surfaces	Self-cleaning function of various plant leaves	Hands-on experiments	Pupils	German [20] German, English [21]
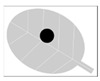	**Lotus-Effect^®^**	Wettability of surfaces	Shape of water droplets	Hands-on experiments	Pupils	German [20] German, English [21]
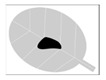	**Lotus-Effect^®^**	Damage of the self-cleaning effect	Effects of damage to the surface properties and destruction of the surface tension of water	Hands-on experiments	Pupils	German [20] German, English [21]
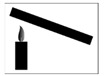	**Lotus-Effect^®^**	Self-cleaning technical surfaces	Production of self-cleaning glass and paper surfaces	Hands-on experiments	Pupils	German, English [21]
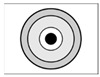	**Velcro^®^**	Velcro target	Construction of a target with various fabrics	Building instruction, Velcro quiz	Pupils	German [22]
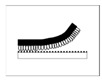	**Velcro^®^**	Application of weight	Pull-off tests in different directions of the hook-and-loop fastener	Hands-on experiments	Pupils	German, English [21]
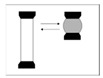	**Fluidic muscle**	Movement quality	Comparison of the Fluidic Muscle and a double-acting cylinder	Hands-on experiments	Pupils	German, English [21]
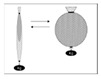	**Balloon muscle**	Bio-inspired actuator	Lifting a weight with balloon, net and cable ties	Building instructions, hands-on experiments	Kindergarten children	German [23]
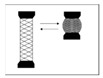	**Meshed actuator**	Plant-inspired actuator	Linear and curved mesh demonstrators	Building instructions, hands-on experiments	Pupils, students	English [24]
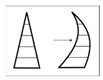	**FinRay Effect**	Self-adapting gripper	Comparison of shape-adjustment of various grippers	Building instruction, hands-on experiments	Pupils	German, English [21]
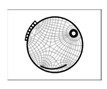	**Lightweight ceiling**	Bone-inspired ceiling	Construction along the main force trajectories	Photoelasticity, hands-on experiments	Pupils	German [25], English Supplementary Material S1
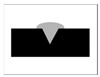	**Self-repairing materials systems**	Wound repair in plants	Self-sealing cells	Hands-on experiments	Pupils	German [26]
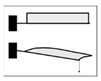	**Flectofin^®^**	*Strelitzia* flower meets architecture	Hinge-less movement in plants and technology	Building instructions, hands-on experiments	Pupils, students	German [19]
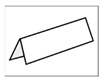	**Foldings**	Folding in nature and technology developed in parallel	2D- and 3D-shapes of basic patterns of folding	Templates for paper folding models	Pupils, students	German [27]
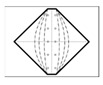	**Plant motions**	Self-actuated paper and wood models	Spatially complex plant movements	Building instructions and templates, hands-on experiments	Pupils, students	English [28]
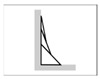	**Method of tensile triangles**	Durable components	Construction of an optimized champagne glass	Photoelasticity, hands-on experiments	Pupils	German, English [21]
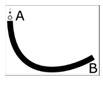	**Evolutionary algorithms**	Brachistochrone problem	Marble run with a curve of fastest descent	Online-experiment EvoBrach, building instruction, hands-on experiments	Students	German [29]
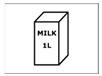	**Evolutionary algorithm**	Optimization of a milk carton	Packaging 1 liter of milk with as little material as possible	Hands-on experiments	Students	German [30]
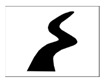	**Plant biomimetics on a stroll**	Guided biomimetics tour in the Botanic Garden	Selection of well-known examples	Simple experiments on biomimetics and biomechanics	All groups	German [31,32]

## Data Availability

All relevant data are included within the paper and its Supplementary Materials file.

## Supplementary Materials

The following is available online at https://www.mdpi.com/article/10.3390/biomimetics6030049/s1, Material S1: Educational module “The bone-inspired ceiling”.
