# Supplementary material for: Biomimetics and Education in Europe: Challenges, Opportunities, and Variety
Source: Biomimetics (Basel). 2021 Aug 4;6(3):49. doi: 10.3390/biomimetics6030049 (PMC8395498; doi:10.3390/biomimetics6030049)
Supplement: Supplementary file 1 [file biomimetics-06-00049-s001.zip › biomimetics-1272774-supplementary.pdf]

biomimetics

Supplementary Material

Biomimetics and Education in Europe: Challenges, Opportunities, and Variety

Olga Speck [1,2,*] and Thomas Speck [1,2]

[1] Plant Biomechanics Group @ Botanic Garden Freiburg, University of Freiburg, D-79104 Freiburg, Germany; olga.speck@biologie.uni-freiburg.de (O.S.); thomas.speck@biologie.uni-freiburg.de (T.S.)
[2] Cluster of Excellence *liv*MatS @ FIT – Freiburg Center for Interactive Materials and Bioinspired Technologies, D-79110 Freiburg, Germany; olga.speck@biologie.uni-freiburg.de (O.S.); thomas.speck@biologie.uni-freiburg.de (T.S.)
* Correspondence: olga.speck@biologie.uni-freiburg.de; Tel.: +49-761-203-2803

Educational module: "The bone-inspired ceiling"

The presented module is based on the development of the bone-inspired concrete ceiling of the Old Zoology Lecture Hall at the University of Freiburg, Germany. This module is suitable for 2 to 3 pupils or students from 15 years onward. For safety reasons, an adult should be present when the gelatin is cooked and poured. The photoelasticity experiment should be carried out in group work or teamwork. The degree of difficulty is "medium". The experiment takes 3 x 45 minutes + 1 night, because the gelatin must cool overnight in the refrigerator.

The following instructions are addressed directly to the pupils and students. The instructions are divided into six part:

1. Information: The bone-inspired ceiling
2. Experiment: Mechanical stress in a gelatin body
3. Observation: Stress lines occur under pressure
4. Solution: Stress distribution depends on the arrangement of the supports
5. Explanation: Changes of optical properties under mechanical deformation
6. Information about photoelasticity

The Bone-inspired Ceiling

Biomimetics in architecture—In recent years, the construction principles of plants and animals have repeatedly proven to be a treasure trove of biomimetic structures. The structure of the fine network of bone trabeculae inside bones has always fascinated architects. The reinforced concrete ceiling of the Old Zoology Lecture Hall at the University of Freiburg (Germany) is a biomimetic lightweight structure. Bone trabeculae were the inspiration for the reinforced concrete ribs of the ceiling structure in the lecture hall. However, how can you determine where these ribs should run? You can carry out an easy photoelastic experiment and make visible the places at which the (main) tensile and compressive forces occur. Have fun cooking a gelatin body and experimenting!

Scientists and architects have always been fascinated by the structure of the fine network of bone trabeculae inside the bones. Like bones, buildings should be both strong and light-weight. When planning the Old Zoology Lecture Hall at the University of Freiburg in 1969, the architect H.-D. Hecker faced the task of designing a bright and circle building with a diameter of 24 meters. The architect was aware of the obvious similarity of the reinforced concrete ceiling to the structure of bones. He found it highly symbolic that zoology students were studying under a bone-inspired ceiling.

Inspired by nature—Bones perfectly combine the seemingly contradictory mechanical properties of being light-weight and stable. They must be stable, because they bear the entire weight of the body; and they must be light-weight, because additional weight consumes unnecessary energy during locomotion. If you look at the longitudinal section of the bone in Figure 1, you can see that it is not solid but consists of a fine network of bone trabeculae. These bone trabeculae are aligned along the stress lines that occur under mechanical loading. Bones are constantly being remodeled and adapted to new mechanical stresses as they arise. Thus, bone material is only found in the loaded areas; whereas in unloaded areas, the bone is broken down again.

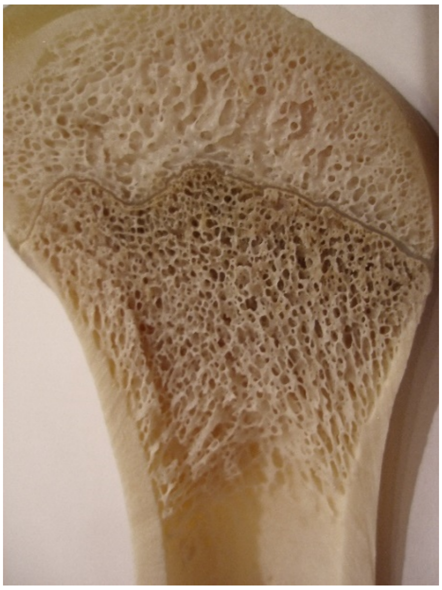

Figure 1: The femur of a lamb in longitudinal section. The fine network of bone trabeculae is clearly visible.

The ribbed ceiling grows—In 1870, the engineer K. Culmann recognized that bone trabeculae follow exactly the course of the theoretical lines of compression and tension.

The Bone-inspired Ceiling

The architect H.-D. Hecker was aware of this relationship and used the principle of isostatic ribs in the ceiling construction of the Old Zoology Lecture Hall at the University of Freiburg. In this context, isostatic means that all ribs are (in the ideal case) subjected to identical mechanical loads. Building material was selectively reinforced along the main stress lines, whereas in other areas, material was saved. The material savings achieved in this way allow weight and costs to be reduced.

Course of reinforced concrete ribs— An inanimate structure with isostatic ribs cannot grow like a bone or adapt to changing environmental conditions. The architect must therefore design the construction to withstand all conceivable loads. Today, civil engineers carry out these highly complex calculations using computer simulations. In the 1960s, however, such computer programs did not yet exist. At that time, the course of the isostatic ribs in the circular ceiling were determined and optimized in models.

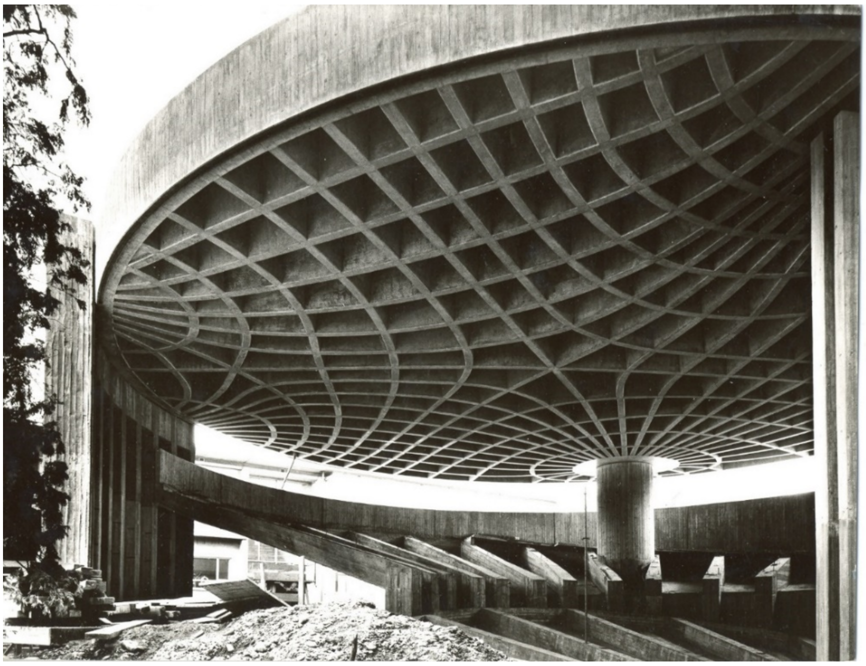

Figure 2: Former zoology lecture hall at the University of Freiburg with its bone-inspired ceiling during construction. © Bruno Krupp, Freiburg and university building authority (Universitäts-bauamt) Freiburg.

Photoelasticity tests—The architect H.-D. Hecker commissioned the Technical University of Karlsruhe to carry out photoelastic tests on a round Plexiglas plate with a diameter of 1.2 meters. Photoelasticity is a common method for visualizing mechanical stresses. In order to optimize statically the course of the reinforced concrete ribs, the engineers analyzed various loading scenarios, such as additional unilateral loads because of snow. Finally, they revised the determined values mathematically and, to a lesser extent, in terms of design. In addition, they made concessions regarding the production of the formwork. The architect used the similarity between bone trabeculae and the ceiling structure to emphasize the function of the Zoology Lecture Hall of the Biology Faculty at the University of Freiburg (Figure 2).

The Bone-inspired Ceiling

Can you see where a structure is exposed to mechanical stress? Yes—quite simply with photoelastic tests. Head to the kitchen to cook a gelatin body and off you go!

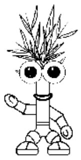	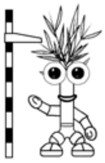	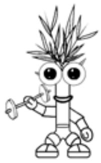	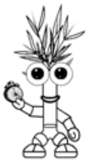
**Working type:** partner or group work of 2 to 3 pupils or students	**Age:** pupils older than 15 years & 1 adult or students	**Degree of difficulty:** medium	**Duration:** 3 x 45 minutes & 1 night

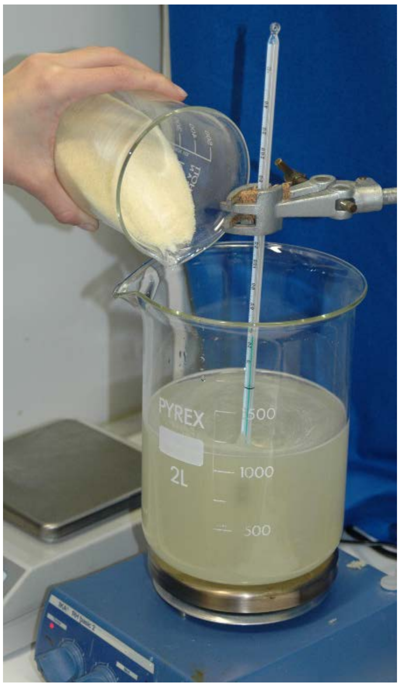

Figure 3: *Preparation of the gelatin with a hot plate and a stirring fish. However, it works just as well with a hot plate and a whisk.*

What you need:

- water
- gelatin in powder form*
- glycerin*
- pot or beaker
- balance
- stove top or hot plate
- whisk or stirring fish
- thermometer
- gloves
- kitchen gloves
- apron
- cake ring (set Ø 25 cm) or various molds (height at least 4 cm)
- flat smooth base for pouring (if using a cake ring, e.g., glass plate)
- 2x Plexiglas sheets 30 cm x 30 cm (thickness not important)
- 2x polarizing foil* 30 cm x 30 cm
- overhead projector or slide viewer
- building blocks

*Notes: Sources of supply and materials used (please see below)

Procedure

1. Preparing the gelatin

For the gelatin body made in a cake ring (Ø 25 cm, height at least 5 cm): Mix 425 g gelatin, 75 g glycerin, and 2 liters of water at about 50-60°C (thermometer!) in a beaker or saucepan on a hot plate or stove top to obtain lump-free gelatin that is as clear as possible without air bubbles (Figure 3). While doing this, mix some of the water with the gelatin and glycerin and then gradually add it to the remaining heated water while stirring constantly (stirring fish/whisk). You will get a better result the longer you stir and heat the solution. The temperature should remain between 50°C and 60°C. If you use a different shape or size than the specified cake ring, you will have to calculate the amount of ingredients that you will need (**notes, see below).

In summary: You use a 20% gelatin mixture, where the dry matter is composed of 15% glycerin and 85% gelatin powder.

2. Filling the molds

Pour the gelatin slowly into the cake ring or mold, preferably without creating bubbles. The gelatin should be at least 3–4 cm high (see Figure 4). Let everything cool **overnight** in a refrigerator and then carefully release the poured gelatin from the mold.

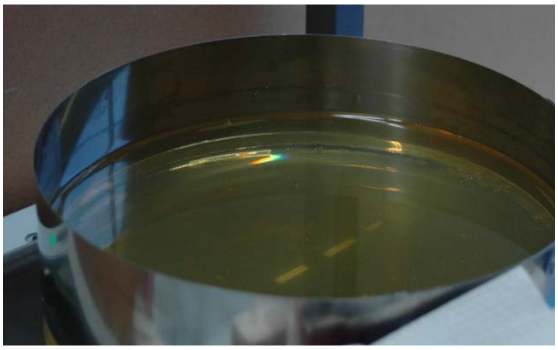

Figure 4: *Cake ring with poured and cooled gelatin shortly before release.*

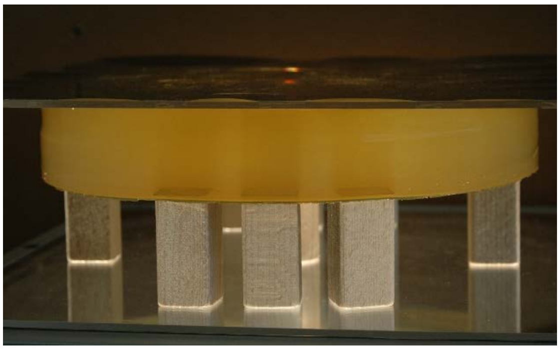

Figure 5: *Experimental arrangement with building blocks under the gelatin body.*

3. Photoelastic test

Lay the prepared polarization foils onto the Plexiglas sheets. The Plexiglass sheets serve to protect the expensive films, so that you can use them repeatedly. Place one of the Plexiglas-supported polarization foils directly onto the switched-on overhead projector.

Now build your "gelatin ceiling" on top of it. Think about an arrangement of building block supports on which you can then carefully place your cast gelatin body (see Figure 5). If the construction is too wobbly, you can also place the supports on top of the gelatin (see Figure 6). Put the second Plexiglas-supported polarization foil on top of your construction. Make sure that the direction of the black lines is perpendicular to the first foil.

Now exert pressure on your construction with the upper Plexiglas-supported foil. The pressure creates mechanical stresses in the gelatin body, and you should see a change in the color of the gelatin body. If not, turn the upper foil by 90° and it will work. If you still do not see any color change, just press a little harder.

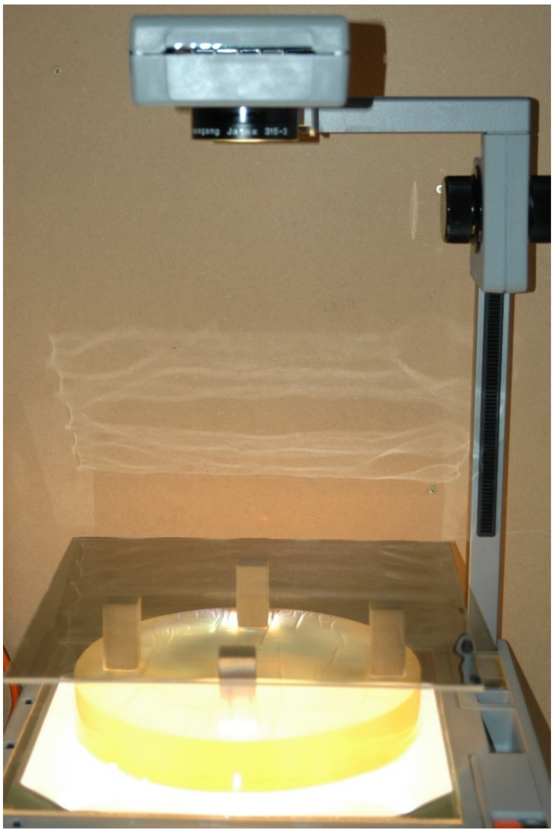

Figure 6: *Experimental set-up consisting of an overhead projector, 2 Plexiglas sheets with polarization foils, gelatin body and building blocks. If the gelatin body is stable enough, the building blocks can also be arranged underneath it.*

***Notes: Sources of supply and materials used**

Polarization foil: our source of supply: e.g., www.astromedia.de
Gelatin: we used: gelatin gold, purest, 180 Bloom
If you use food gelatin from the supermarket, then you may have to adjust the recipe.
Glycerin: we used: Rotipuran®, > 99.5%, p.a., anhydrous.

Tips:

- You can pour all kinds of shapes from gelatin, and you can melt the gelatin bodies a few times and reuse them before the gelatin spoils.
- Cake rings are available on the Internet in many different shapes.
- Be careful when opening the cake ring. They sometimes have sharp edges. They are under tension when closed and pop open when released. Put on kitchen gloves as a precaution.

**** Notes: Calculation for a cake ring with a different diameter**

If you want to use a different mold or size than the cake ring given in the recipe above, you can calculate the ingredients like this:

- Water = 0.8 x volume of the mold
- {Gelatin & glycerin} = 0.2 x volume of the mold
- Volume of gelatin powder = 0.85 x {gelatin & glycerin}
- Volume of glycerin = 0.15 x {gelatin & glycerin}

The following tips for calculation should help:

- Volume of a cylinder (like your cake ring) = $\pi \cdot radius^2 \cdot height$ [cm^3]
- Remember: 1 liter = 1 dm^3; if you use radius and height with the unit dm (= 10 cm) to calculate the volume, you will get your result in liters. You can easily measure these volumes with a measuring cup.

The Bone-inspired Ceiling

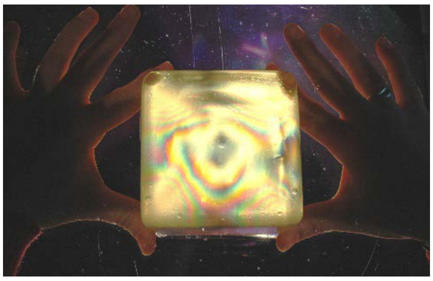

Change with pressure

Describe how the appearance and color of your gelatin body changes when you first apply weak pressure and later apply strong pressure.

Course of the stress lines

Describe where the colored stress lines are densest and where they are most pronounced. Can you give reasons?

Changed arrangement of the supports

Describe how the appearance of the mechanical stress lines changes when you change the number and arrangement of the supports (building blocks).

The Bone-inspired Ceiling

Change with pressure

Describe how the appearance and color of your gelatin body changes when you first apply weak pressure and later apply strong pressure.

If sufficient pressure is applied to the gelatin, colored lines can be seen within the gelatin body (Figure 7). The more the pressure is applied, the more of these colored lines become visible and their color and position can change.

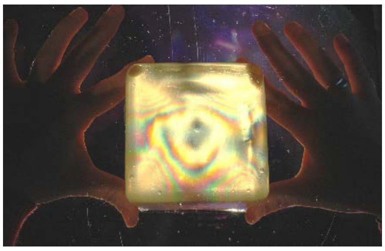

Figure 7: *Square gelatin body with simultaneous pressure at all four corners.*

Course of the stress lines

Describe where the colored stress lines are densest and where they are most pronounced. Can you give reasons?

The stress lines condense toward the supports where they are the most pronounced (Figure 8). ***Reasons:*** *The weight of the support and the gelatin are concentrated locally onto a small area. As a result, the mechanical stress is particularly high at this point*.

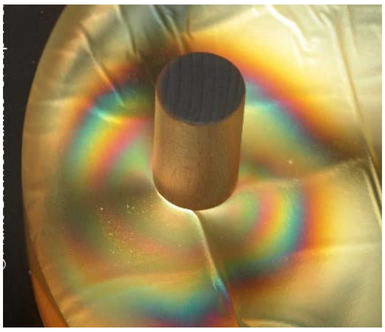

Figure 8: *Stress lines around a circular support.*

Changed arrangement of the supports

Describe how the appearance of the mechanical stress lines changes when you change the number and arrangement of the supports (building blocks).

Depending on the number and arrangement of the supports, the colored lines are differently located and differently pronounced. The mechanical stress is distributed differently (Figures 9 and 10).

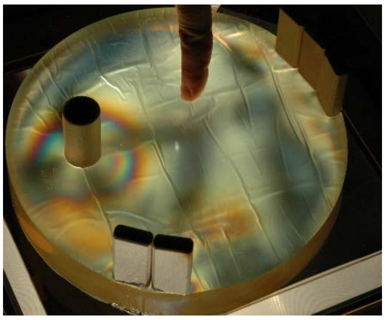

Figure 9: *Arrangement of the supports similar to the ribbed ceiling of the Zoology Lecture Hall.*

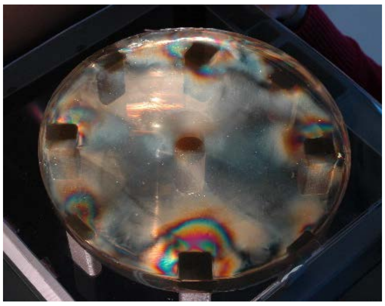

Figure 10: *The changed arrangement of the supports leads to a different distribution of the stress lines.*

Change with pressure

The optical properties of the gelatin change as a result of the mechanical stress. The mechanical stress distribution in the model becomes visible with the help of the two polarization foils. Colored, bright, and black lines are created.

Course of the stress lines and **Changed arrangement of the supports**

The distribution and magnitude of the mechanical stresses in the gelatin body can be observed by means of the course of the stress lines. Black lines indicate the main direction of stress in the gelatin model. The bright and colored lines and areas make high mechanical stresses visible.

For more information on the theoretical background of photoelasticity, see the "Photoelasticity" worksheet.

Photoelasticity

Use of photoelasticity

Photoelasticity was previously a common method for visualizing mechanical stresses without determining absolute values. Photoelastic images show the direction of the principal stresses and indicate the locations of low stress areas and areas with high local stresses in the model. Today, these stress patterns are simulated in the computer.

The optical effect

Mechanical stresses influence the optical properties of transparent materials (e.g., Plexiglas, acryl glass, gelatin). This optical effect is used in photoelasticity to visualize the stress distribution in mechanically stressed bodies. Based on the stress distribution, we can judge where stress concentrations occur and where the body needs to be reinforced.

Gelatin models

For photoelastic investigations, a gelatin model is made in the shape of the work piece to be examined. Under mechanical stress this model exhibits dark, bright, and colored zones when illuminated by linearly polarized light.

Birefringent material

Photoelastic studies can only be performed with material that is birefringent, such as gelatin or Plexiglas (Figure 11) and acrylic glass. Birefringence arises when the speed of propagation of light in the material is different in different directions.

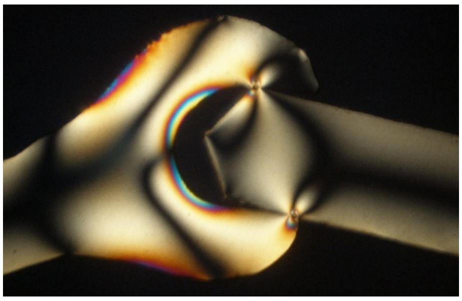

Figure 11: *Photoelastic image of a Plexiglas model of a spanner and nut under mechanical stress showing dark, bright, and colored zones.*

The key: polarized light

The refractive index indicates how strongly light is refracted in a medium such as gelatin. Since mechanical stresses have various effects in different directions, the changes in refractive index vary in different directions when mechanical stress is applied. The light is no longer refracted to the same extent in all directions. However, since our eye is not able to distinguish the directions of oscillation of a light beam, a trick must be used to make the changes in direction visible: therefore polarized light is used.

Photoelasticity

Refractive index: $n = \frac{c}{v}$

n = refractive index [dimensionless]
c = speed of light in vacuum [m/s]
n = modified speed of light in medium [m/s]

A beam of light can be described in simplified terms as a superposition of many waves with different levels of oscillation, all propagating in the same direction.

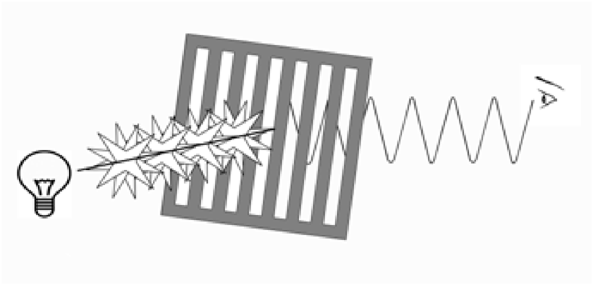

Figure 12: *The polarization filter only allows the permeation of light in one oscillation plane.*

Linearly polarized light consists exclusively of waves with the same plane of oscillation. To "produce" polarized light, a polarization filter is used on which parallel black lines run next to each other. The lines are so close together that only light waves with one direction of oscillation can pass through. All other waves are filtered. This polarizer produces linearly polarized light (Figure 12).

Polarizer and analyzer

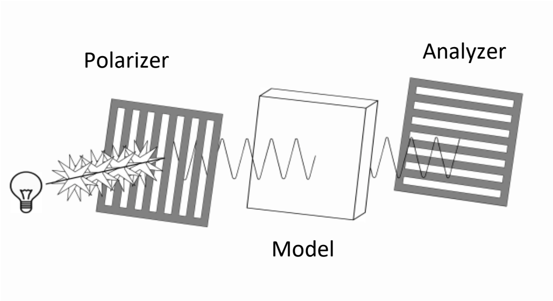

Figure 13: *Photoelastic studies with unloaded gelatin body.*

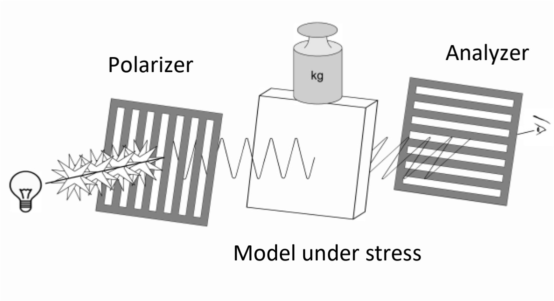

Figure 14: *Photoelastic studies with gelatin body under stress.*

Photoelasticity

If a mechanically unloaded gelatin model is placed between the polarizer and the analyzer (rotated by 90°), the polarization of the light is unchanged. It remains dark (Figure 13).

When the gelatin model is mechanically stressed, the birefringent property of the material changes the situation. When light enters the gelatin body under stress, the linearly polarized light becomes elliptically polarized. This means that the linearly polarized light is split into two perpendicular components oscillating in the direction of the main stresses. Now the light also contains components in the direction of the analyzer and the mechanically stressed areas become visible as colored components (isochromats) (Figure 14). Isochromats provide information about the anisotropy ratios (different properties in the spatial directions) in the model. The black lines and areas (isoclines) directly show the course of the principal stresses occurring in the model.
